# Sex-specific responses to slow progressive pressure overload in a large animal model of HFpEF

**DOI:** 10.1152/ajpheart.00374.2022

**Published:** 2022-09-02

**Authors:** Deborah M. Eaton, Remus M. Berretta, Jacqueline E. Lynch, Joshua G. Travers, Ryan D. Pfeiffer, Michelle L. Hulke, Huaqing Zhao, Alexander R. H. Hobby, Giana Schena, Jaslyn P. Johnson, Markus Wallner, Edward Lau, Maggie P. Y. Lam, Kathleen C. Woulfe, Nathan R. Tucker, Timothy A. McKinsey, Marla R. Wolfson, Steven R. Houser

**Affiliations:** ^1^Cardiovascular Research Center, Lewis Katz School of Medicine, Temple University, Philadelphia, Pennsylvania; ^2^Department of Cardiovascular Sciences, Lewis Katz School of Medicine, Temple University, Philadelphia, Pennsylvania; ^3^Department of Physiology, Lewis Katz School of Medicine, Temple University, Philadelphia, Pennsylvania; ^4^Department of Thoracic Medicine and Surgery, Lewis Katz School of Medicine, Temple University, Philadelphia, Pennsylvania; ^5^Department of Pediatrics, Lewis Katz School of Medicine, Temple University, Philadelphia, Pennsylvania; ^6^Center for Translational Medicine, Lewis Katz School of Medicine, Temple University, Philadelphia, Pennsylvania; ^7^CENTRe: Consortium for Environmental and Neonatal Therapeutics Research, Lewis Katz School of Medicine, Temple University, Philadelphia, Pennsylvania; ^8^Division of Cardiology, Department of Medicine, University of Colorado Anschutz Medical Campus, Aurora, Colorado; ^9^Consortium for Fibrosis Research & Translation, University of Colorado Anschutz Medical Campus, Aurora, Colorado; ^10^Masonic Medical Research Institute, Utica, New York; ^11^Cardiovascular Disease Initiative, Broad Institute of MIT and Harvard, Boston, Massachusetts; ^12^Center for Biostatistics and Epidemiology, Department of Biomedical Education and Data Science, Lewis Katz School of Medicine, Temple University, Philadelphia, Pennsylvania; ^13^Division of Cardiology, Medical University of Graz, Graz, Austria

**Keywords:** heart failure with preserved ejection fraction, hemodynamics, hypertrophy, sex-based differences, single-nucleus RNA sequencing

## Abstract

Approximately 50% of all heart failure (HF) diagnoses can be classified as HF with preserved ejection fraction (HFpEF). HFpEF is more prevalent in females compared with males, but the underlying mechanisms are unknown. We previously showed that pressure overload (PO) in male felines induces a cardiopulmonary phenotype with essential features of human HFpEF. The goal of this study was to determine if slow progressive PO induces distinct cardiopulmonary phenotypes in females and males in the absence of other pathological stressors. Female and male felines underwent aortic constriction (banding) or sham surgery after baseline echocardiography, pulmonary function testing, and blood sampling. These assessments were repeated at 2 and 4 mo postsurgery to document the effects of slow progressive pressure overload. At 4 mo, invasive hemodynamic studies were also performed. Left ventricle (LV) tissue was collected for histology, myofibril mechanics, extracellular matrix (ECM) mass spectrometry, and single-nucleus RNA sequencing (snRNAseq). The induced pressure overload (PO) was not different between sexes. PO also induced comparable changes in LV wall thickness and myocyte cross-sectional area in both sexes. Both sexes had preserved ejection fraction, but males had a slightly more robust phenotype in hemodynamic and pulmonary parameters. There was no difference in LV fibrosis and ECM composition between banded male and female animals. LV snRNAseq revealed changes in gene programs of individual cell types unique to males and females after PO. Based on these results, both sexes develop cardiopulmonary dysfunction but the phenotype is somewhat less advanced in females.

**NEW & NOTEWORTHY** We performed a comprehensive assessment to evaluate the effects of slow progressive pressure overload on cardiopulmonary function in a large animal model of heart failure with preserved ejection fraction (HFpEF) in males and females. Functional and structural assessments were performed at the organ, tissue, cellular, protein, and transcriptional levels. This is the first study to compare snRNAseq and ECM mass spectrometry of HFpEF myocardium from males and females. The results broaden our understanding of the pathophysiological response of both sexes to pressure overload. Both sexes developed a robust cardiopulmonary phenotype, but the phenotype was equal or a bit less robust in females.

## INTRODUCTION

Heart failure with reduced and preserved ejection fraction (HFrEF and HFpEF) are the two major forms of heart failure (1). HFpEF is less well understood than HFrEF, but its prevalence is growing by 10% per decade and there are few clinical options to help patients suffering from this syndrome (2). Patients with HFpEF are more likely to be of an older median age, more likely to be females (3), and have a higher frequency and severity of noncardiac comorbidities including renal dysfunction, metabolic syndrome, and obesity compared with patients with HFpEF (1). The respective contribution of each of these pathological stressors to the HFpEF phenotype is not well known. What is clear is that therapies such as angiotensin-converting enzyme inhibitors, angiotensin receptor blockers, mineralocorticoid receptor antagonists, angiotensin receptor-neprilysin inhibitors, and β-blockers have failed to produce positive outcomes in clinical trials for patients with HFpEF while having positive effects in patients with HFpEF (4). EMPEROR-Preserved, a randomized clinical trial comparing empagliflozin (sodium glucose cotransporter 2) versus placebo in patients with HF with an EF greater or equal to 40%, was recently completed and produced positive outcomes giving hope for new treatments (5). However, HFpEF is not a disease with one cause and instead represents a syndrome with a spectrum of causes and phenotypes (6). Importantly, hypertension (pressure overload, PO) is a common finding in patients with HFpEF (7). Given the complexity of this syndrome, having a spectrum of animal models capturing different aspects of HFpEF will be essential in better describing the pathophysiological changes occurring in the disease progression.

We previously performed an in-depth characterization of the effects of slow progressive pressure overload (PO) on the cardiopulmonary phenotype of male felines (2). A constrictive (band) was loosely placed around the ascending aorta of small, immature animals just before they enter a rapid period of growth. As animals grow, they slowly develop aortic constriction causing slow progressive pressure overload (PO) of the left ventricle (LV). This stress induces a robust disease phenotype with cardiopulmonary impairments. The model recapitulates key clinical features necessary to secure a guideline-based HFpEF diagnosis, such as signs and symptoms of HF, elevated NH_2_-terminal-prohormone B-type natriuretic peptide (NT-proBNP), left atrium (LA) enlargement and dysfunction, LV hypertrophy and diastolic dysfunction, and pulmonary hypertension, which is present in up to an estimated 80% of patients with HFpEF (1, 8). These phenotypic features correspond with the American College of Cardiology/American Heart Association (ACC/AHA) stage B/C of HF (2, 9–11).

The animals used in this model are young and have no overt comorbidities, which allow for evaluation of the effects of slowly progressing PO without confounding factors. Furthermore, the feline model has key physiological features identical to humans, such as a long action potential duration and the predominance of β-myosin heavy chain, which should increase the possible translation of findings to at least some fraction of patients with HF (12, 13).

Given the increased HFpEF prevalence in females (14), the present study asked whether female animals develop a more robust cardiopulmonary phenotype with pressure overload than seen in males and, if so, begin to define its cellular and molecular bases. Our results show that the cardiopulmonary phenotype of females with PO is similar but often less robust than observed in males. These results suggest that PO, by itself, is not sufficient to induce major differences in sex-specific HFpEF phenotypes in this animal model.

## METHODS

### Study Design

**Figure 1.**
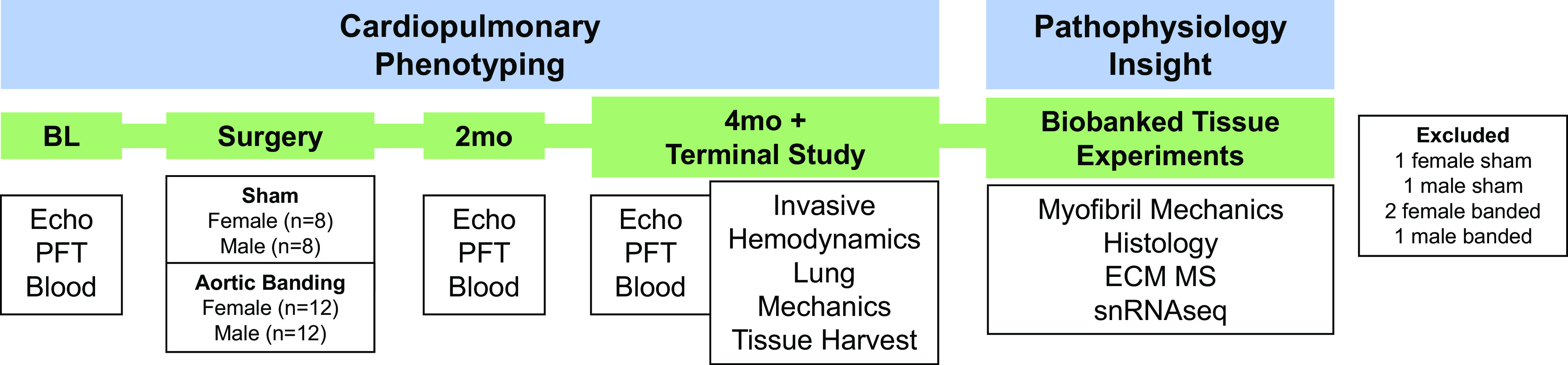
Study design. Male and female felines (age 2 mo) underwent baseline echocardiography, pulmonary function testing, and blood collection. Animals then underwent either sham surgery or aortic banding, and then serial echocardiography and pulmonary function testing was performed at 2 and 4 mo postsurgery. At 4 mo, terminal studies were performed to record invasive hemodynamics, lung mechanics, and then harvest tissue for biobanking. To further characterize the phenotype, histological analyses, extracellular matrix mass spectrometry (ECM MS), and single-nucleus RNA sequencing (snRNAseq) were performed. BL, baseline; Echo, echocardiography; PFT, pulmonary function testing.

All animal procedures were approved by the Lewis Katz School of Medicine Temple University Institutional Animal Care and Use Committee. The following protocol has been extensively described by our laboratory (2, 15). The experimental protocol is outlined in Fig. 1. A total of 20 male and 20 female short-hair felines (aged 2 mo; ∼1.0 kg; Marshall Bioresources, Waverly, NY) were included in the study. It is important to note that none of the male animals described in this study were included in any recent reports from our laboratory (2, 15). Animals that underwent the aortic banding procedure will be referred to as male banded and female banded (male, *n* = 12; female, *n* = 12), while animals that underwent sham surgery will be referred to as male sham and female sham (male, *n* = 8; female, *n* = 8). Felines underwent baseline echocardiography, pulmonary function testing, and blood collection. Animals were randomized into each group (aortic banding or sham) based on their body weight recorded at the time of baseline (BL) echocardiography to ensure there was no body weight difference between groups. Either sham surgery or aortic banding was performed, and animals then underwent serial echocardiography and pulmonary function testing at 2 and 4 mo postsurgery. At 4 mo, terminal studies were performed to measure hemodynamic properties, lung mechanics, and then harvest tissue for biobanking. To further characterize the phenotype, histological analyses, extracellular matrix (ECM) MS, and single-nucleus RNA sequencing (snRNAseq) were performed.

Investigators were blinded to sex and condition until all analyses were completed. After all data were analyzed, one male sham and one female sham animal were excluded from analyses because of signs of cardiac disease [echocardiography (ECHO) parameters outside of range for healthy animal], and one male band and two female bands were excluded because of incomplete hemodynamic measurements. No animals died prematurely during the study due to surgery or the aortic banding causing immediate pressure overload.

### Serial Transthoracic Echocardiography

Serial echocardiography measurements were acquired using the Vivid q Vet Premium BT′12 with a 12S-RS sector probe. Animals were sedated with an intramuscular injection of alfaxalone (1 mg/kg), butorphanol (0.5 mg/kg), and midazolam (0.5 mg/kg). After venous blood collection and before beginning the echo assessment, ivabradine (0.3 mg/kg) was administered (intravenously via cephalic vein) to decrease the heart rate to around 150 beats/min (16). Blinded analysis was performed offline using the EchoPAC SW v201 software. Left ventricular (LV) wall thickness was measured at end-diastole by calculating the mean of LV anterior ventricular septum and posterior wall diameter. The left atrial aortic root ratio (LA/Ao) was measured in early ventricular diastole using the first frame after aortic ejection from the right parasternal short-axis view (17). LA volume (LAV) was calculated as described in humans (18) from a right parasternal long-axis view, using the Simpson’s rule at end-systole and end-diastole. LA function, assessed by LA volumes, was calculated according to atrial function studies in humans (19). Changes in LAV were expressed as ejection fraction [LA − EF = (LAV_max_ − LAV_min_)/LAV_max_] (20). LV end-diastolic diameter and ejection fraction (EF) were measured in B-mode from a right parasternal short-axis view. Diastolic function was assessed using pulsed-wave Doppler (PW) imaging. With an apical four-chamber view, transmitral inflow velocities were recorded by setting the sample volume in the mitral orifice close to the tip of the mitral leaflets. Spectral waveforms were analyzed for peak early- and late-diastolic transmitral velocities (*E* and *A* waves). If there was any fusion of the *E*/*A* waves, that time point was excluded for the specific animal.

### Aortic Banding

For surgery, animals were sedated with ketamine (25 mg/kg) and acepromazine (0.1 mg/kg), intubated, and mechanically ventilated (Narkomed 2b). During the procedure, surgical (plane) anesthesia was maintained using 1%–2% isoflurane mixed with 100% oxygen, and animals received constant heat support during the surgery and postoperative period. All surgical procedures were performed under sterile operating conditions. For both sham and banded animals, a 2–4 cm skin incision was made between the 3rd to 4th intercostal space, extending through the intercostal muscles. After the pericardium was identified and nicked to open, the aorta was dissected from the pulmonary artery to place the preshaped band around the ascending aorta. The next step in the surgery is essential in the development of the model. The band must be tied down loosely around the ascending aorta so slow progressive overload can occur as the kitten rapidly grows over the next 4 mo. If the band is tied down too tightly during surgery, it will cause an immediate pressure overload resulting in a more dilated phenotype.

### Hemodynamic Studies and Pulmonary Function Assessment

At 4 mo postsurgery, invasive hemodynamic studies were performed. Induction was performed using intravenous pentobarbital sodium (20 mg/kg) and maintained with 10 mg/kg/h sodium pentobarbital for the duration of the study. Intravenous pancuronium bromide (0.1 mg/kg/h), a paralytic agent, was administered so during the study animals had comparable intrathoracic pressure profiles and were not breathing spontaneously. Animals were then intubated orally with a cuffed endotracheal tube (3.0 mm, Medline, Industries, IL), and mechanically supported with pressure sensitive volume guaranteed ventilation (Babylog 8000 plus, Dräger Lubeck, Germany). All animals were initially ventilated with the same settings: ${\text{FI}}_{{\text{O}}_{2}}$ = 1, peak inspiratory pressure = 10 cmH_2_O, positive end expiratory pressure = 3 cmH_2_O, inspiratory flow = 4 L/min, inspiratory time = 0.66 s, expiratory time = 3 s. Data generated by the integrated pulmonary mechanics module using airway manometry and pneumotachography as fitted to the equation of motion were manually recorded. Arterial and venous blood gas sample analysis was used to guide ventilation to maintain the $Pa_{{\mathrm{CO}}_{2}}$ within 25–35 mmHg. This was achieved by keeping the tidal volume range to 6–8 mL/kg and adjusting peak inspiratory pressures and respiratory phase timing as needed while keeping all other ventilator settings consistent. Animals were infused intravenously with a balanced crystalloid infusion (Ringer-Lactate) at a fixed rate of 10 mL/kg/h for the entire study and body temperature was kept between 38 and 39°C using a heating pad with continuous ECG and $Sp_{O_{2}}$ monitoring. The following vessels were instrumented:

- Right femoral artery: 2-F pressure catheter (SPR-320 Mikro-Tip)
- Left carotid artery: 2-F pressure catheter (SPR-320 Mikro-Tip).

All catheters were placed using fluoroscopic guidance. Data were acquired using PowerLab and LabChart Pro 8.1.5 (ADInstruments) and analyzed offline using LabChart Pro 8.1.5 (ADInstruments).

After all instrumentation was completed, animals were given at least 30 min to stabilize. Fractional flow reserve (FFR) module (Quantien, St. Jude Medical Inc.) was used to invasively measure transaortic pressure gradients across the band. Steady-state hemodynamics were recorded at end-expiration with a steady positive end-expiratory pressure (PEEP) of 3 cmH_2_O, this minimized respiration-induced changes of intrathoracic pressure.

### Heart Procurement and Processing

After completing the hemodynamic study, a cardiectomy was performed to remove the heart, which was then rinsed and weighed. The aorta was cannulated so the coronary arteries could be flushed using cold Krebs–Henseleit Buffer. The bottom portion of the heart was then cut so samples could be flash frozen for molecular studies. The hearts were then switched to gravity perfusion with 10% formalin at a mean arterial pressure (100 mmHg) and collateral vessels from where the apex was cut were clipped to generate back pressure. After satisfactory formalin perfusion, the hearts were immersed in 10% formalin until the tissue was cut and processed. The heart was cut on a short-axis plane, starting at the apical end and continuing up to the base. These short-axis sections were then cut in half (lateral and septal wall) and shipped to AML Laboratories for processing and embedding paraffin. For slides, 5 µm tissue sections from six different levels from each sample were mounted (AML Laboratories).

### Heart Histology

To determine the percentage of fibrosis, paraffin-embedded LV samples were stained with Masson’s Trichrome (Sigma-Aldrich; St. Louis, MO). Cytoplasm and muscle fibers are stained red, while collagen (fibrosis) is stained blue. Endocardial and epicardial regions of the heart were imaged separately so they could be quantified for comparison. Sixty-eight slides (34 lateral wall, 34 septal wall) from 34 animals (banded male: *n* = 11, sham male: *n* = 6, banded female: *n* = 10, sham female: *n* = 7) were analyzed to obtain representative data from the lateral and septal walls. The stained slides were imaged at ×10 magnification using a Nikon Eclipse Ti microscope with an attached bright-field camera (Nikon Inc.; Mellvile, NY) and analyzed using NIH ImageJ FIJI software with color threshold analysis. A total of 1,090 pictures were analyzed. The percentage of fibrotic tissue was determined as the collagen positive tissue out of the total stained LV tissue.

To quantify myocyte cross-sectional area, LV tissue sections were stained for wheat germ agglutinin (WGA; Life Technologies, Eugene, OR) and nuclei were labeled with 4′,6-diamidino-2-phenylindole (DAPI, Millipore; Billerica, MA). Images were taken using a Nikon Eclipse T1 confocal microscope (Nikon Inc.; Mellvile, NY). Myocyte cross-sectional area was measured using NIH ImageJ FIJI software as previously described (10). A total of 36,401 myocytes were counted from 27 animals (banded male: *n* = 9, sham male: *n* = 6, banded female: *n* = 9, sham female: *n* = 7). The mean cross-sectional area was reported for each animal.

### Myofibril Isolation

Myofibrils were isolated as described previously (21, 22). A small section of flash-frozen left ventricle (LV) was cut into thin slices and bathed in 0.05% Triton X-100 in Linke’s solution with protease inhibitors (50 mM Tris, 100 mM KCl, 2 mM MgCl_2_, 1 mM EGTA, pH 7.0; 10 μM leupeptin, 5 μM pepstatin, 200 μM phenyl-methylsuphonylfluoride, 10 μM E64, 500 μM NaN_3_, 2 mM dithioerythritol) overnight at 4°C overnight. Skinned tissue was washed three times in rigor solution and resuspended in bath solution (pCa 9.0; 100 mM Na_2_EGTA; 1 M potassium propionate; 100 mM Na_2_SO_4_; 1 M MOPS; 1 M MgCl_2_; 6.7 mM ATP; and 1 mM creatine phosphate; pH 7.0) with protease inhibitors and homogenized at speed 7 for 10 s three times (Tissue Tearor). Myofibril mechanics measurements were completed.

### Myofibril Mechanics

Mechanical measurements were completed on isolated myofibrils using the fast solution switching method (23, 24). Briefly, a small bundle of myofibrils were mounted between two microtools at 15°C in relaxing solution (pCa 4.5). One microtool was attached to a motor that produces rapid length changes (Mad City Labs) and the second microtool was a calibrated cantilevered force probe (9.43 μm/μN). Myofibril length was set ∼2.19 µm and average sarcomere length and myofibril diameter were measured using ImageJ. Mounted myofibrils were activated by solutions of different pCa (6 concentrations; pCa 6.0, 5.8, 5.6, 5.4, 4.5) (24, 25). Data were collected and analyzed using a customized LabView software. Measured mechanical and kinetic parameters were defined as follows: resting tension (mN/mm^2^)—myofibril basal tension in fully relaxing condition; maximal tension (mN/mm^2^)—maximal tension generated at calcium activation; the rate constant of tension development following calcium activation (kACT); the rate constant of tension redevelopment following a release-restretch applied to the activated myofibril (kTR) (26); rate constant of linear phase relaxation (kREL, LINEAR)—the slope of the linear regression normalized to the amplitude of relaxation transient, duration of linear relaxation (tREL, LINEAR)—measured from onset of solution change to the beginning of the exponential force decay, the rate constant of the final exponential phase of force decline (kREL, EXP). Ca^2+^ sensitivity was determined by measuring maximal force development over six concentrations of pCa and normalizing submaximal tension (*P*) to tension generated at pCa 4.5 (*P0*).

### NT-proBNP

Blood was collected via cephalic vein puncture at BL and 4 mo postbanding in spray-coated K2EDTA vacutainers, microcentrifuged at 1,300 rpm for 10 min, and stored at −80°C. Samples were then sent to Idexx Laboratories (Memphis, TN) for NT-proBNP analysis (banded male: *n* = 11, sham male: *n* = 7, banded female: *n* = 10, sham female: *n* = 7).

### Serial Pulmonary Function Testing

The protocol for pulmonary function testing (PFT) has been used and described in the feline model previously (2, 15). Pulmonary mechanics were assessed using tidal volume and transpulmonary pressure measurements acquired from spontaneously breathing animals using a clinical device (PeDS-LAB, MAS, Hatfield, PA). To measure tidal volume, a pneumotachograph is integrated into a facemask and transpulmonary pressure is measured from the difference between airway and esophageal pressure. To measure esophageal pressure, the end of a soft esophageal balloon catheter was connected to a differential pressure transducer. These techniques are used clinically in nonsedated human preterm neonates (as early as first few days of life) and are well tolerated.

### Lung Procurement and Processing

After the heart was procured as described earlier, the pulmonary artery was isolated and cannulated as previously reported (2, 15). The pulmonary vasculature was then perfused with cold sterile 0.9% saline ≤15 mmHg until the perfusate ran clear. The left and right lungs were then identified, isolated, and dissected according to a predetermined matrix to support unbiased sampling procedures (27, 28). Tissue samples (2–∼1 cm^3^) from the right lower lobe were stored in buffered formalin saline, washed using PBS, and then stored in ethanol until processed for cutting slides.

### Lung Histology

All analyses were performed in a blinded fashion to modification and gender. The following histology protocols were used previously for the feline model (2, 15). For histological investigation, embedded lung samples were sectioned at 500 μm intervals. In total, 5 × 5 μm sections from each sample were slide mounted and stained with hematoxylin and eosin. Lung sections were first visualized using transparent grid matrix at low power (×100) and then switched to higher power (×400) to randomize selection of the area for analyses. Every tenth grid region was imaged and digitized to eliminate bias during sampling. Morphometric image analysis (Image Pro Plus, Silver Spring, MD) using customized algorithms free of geometric assumptions were used to assess the expansion index (ratio of volume of gas exchange/parenchymal space) using densitometry, count the number of open gas exchange units per fixed field size using a modified version of the radial alveolar count method, measure alveolar area (∼1,000 alveoli), and thickness of the alveolar-capillary membrane (∼100 alveoli; 4 measurement points/alveolus) (27, 29, 30). Perivascular cuff quantification was performed using modified version of a method described by Lowe et al. (31). Images were digitized and visualized at ×100 magnification through an overlaying grid matrix. Using NIH Image J, the outer border of all vessels in the digitized fixed field was traced and the external diameter of the vessels was measured. The outer border of all vessels ≤500 μm and the perimeter of the corresponding perivascular cuff surrounding each of the vessels were traced. The area of the cuff relative to the area of the vessel was analyzed as a function of group. For each histological parameter reported for the lungs, the mean value was reported for each animal.

### Extracellular Mass Spectrometry

The composition of the cardiac extracellular matrix (ECM) was assessed by quantitative mass spectrometry as described previously (32). Briefly, matrix proteins were enriched from ∼25 mg of frozen cardiac tissue using the Compartment Protein Extraction Kit (EMD Millipore 2145) (33). Starting tissue mass was as follows: Male Sham 25.88 ± 0.55 mg; Female Sham 26.08 ± 1.30 mg; Male Band 25.95 ± 0.56 mg; and Female Band 26.85 ± 0.81 mg. Tissue was homogenized and incubated in a series of buffers to sequentially extract the cytosolic, nuclear, membrane, and cytoskeletal fractions. The ECM-enriched pellets were then resuspended in triethylammonium bicarbonate (TEAB) containing 8 M urea and 10 mM tris(2-carboxyethyl)phosphine (TCEP), followed by alkylation of free cysteines using iodoacetamide. Samples were deglycosylated with PNGaseF and digested into peptides using endoproteinase LysC and MS-grade trypsin. The peptide samples were then subjected to isobaric labeling with tandem mass tags (TMTs). The digested and TMT-labeled peptides were subjected to high-pH reversed-phase fractionation and reconstituted in 0.1% formic acid.

Fractions collected from the high-pH reversed-phase fractionation were further separated using online second-dimension low-pH reversed-phase chromatography via an Easy-nLC 1200 nanoflow-UHPLC system (Thermo Scientific) on an EasySpray C18 column (PepMap RSLC C18, 3 μm particle, 100-Å pore; 75 μm × 15 cm dimension; Thermo Scientific) held at 45°C. The typical solvent gradient was 0–105 min: 0–40% B; 105–110 min: 40–70% B; 110–115 min: 70–100% B; 115–120 min: 100% B; 300 nL/min; Solvent A: 0.1% formic acid in water; Solvent B: 80% acetonitrile in water, 0.1% formic acid. Each high-pH fraction (3 μL) was injected by the autosampler on the Easy-nLC 1200 nanoflow-UHPLC system.

The eluent from the Easy-nLC system was analyzed using a Q-Exactive HF Hybrid Quadrupole-Orbitrap mass spectrometer (Thermo Scientific) coupled online to the nanoflow-UHPLC through a Thermo EasySpray ion source. Mass spectrometry signals were acquired in positive mode with common instrument parameters, typically each MS1 full scan was acquired at 60,000 resolving power in profile mode from *m*/*z* 300 to 1,650, followed by data-dependent Top15 acquisition of MS2 scans at 60,000 resolving power. MS2 isolation window was 1.4. The normalized collision energy was set to stepped normalized collision energy at 27, 30, 32.

Acquired mass spectra were converted to .mzML format using ProteoWizard MSConvert. Database searches were performed using Comet (v.2020.01 rev. 3) (34) with a concatenated decoy search against a *Felis catus* proteome database (Swiss-Prot and TrEMBL) retrieved on August 13, 2021 from http://uniprot.org (35). The database search results were postprocessed using Percolator (Crux version 4.0) (36) and peptides identified at 5% false discovery rate (FDR) were accepted for quantification. TMT reporter ion intensities were extracted using an R script (37) and differential expression analysis performed with the aid of limma v.3.49.0 in R (38). Multiple testing correction for protein quantification was performed using the Benjamini–Hochberg procedure (91). The Naba Matrisome Project (http://matrisome.org), a comprehensive database of the ECM proteome composition, was used for the annotation of core-matrisome and matrisome-associated proteins (39).

### Single-Nuclei RNA Sequencing Analysis

We isolated nuclei from snap-frozen ventricular tissue from 15 animals (8 males and 7 females) representing banded and sham operations as previously described (40). Molecularly barcoded single nuclei emulsions were generated using the 10× Genomics Chromium Controller v3.1 NextGEM Single Cell 3′ Kit, and libraries were constructed according to the manufacturer’s protocol. Libraries were multiplexed for sequencing on Novaseq6000 SP flow cells. Sample sets from each condition were multiplexed on the same lane to minimize batch effects.

### Data Processing

The raw base call sequencing files were demultiplexed and the reads were aligned to the feline genome (*Felis_catus_9.0* build 104) using 10× Genomics Cellranger mkfastq (6.0.0). Reads were trimmed using Cutadapt (41) (v.2.8) with default parameters to remove homopolymers (A30, T30, G30, and C30) and the template switch oligo ( CCCATGTACTCTGCGTTGATACCACTGCTT) and its complement ( AAGCAGTGGTATCAACGCAGA GTACATGGG). Count matrices were then generated using CellRanger Count [v.12 within the Cumulus (42) workflow].

Postprocessing and analysis of the data were performed in the Terra Platform (app.terra.bio) using SCANPY v1.7.1 (43). Each sample was inspected for mapping quality based on the number of mapped reads per cell, the percentage of mapped mitochondrial reads, and the shape of the unique molecular identifier (UMI) decay curve, of which all samples demonstrated adequate mapping quality. The samples were then filtered for ambient RNA byproducts of nuclear isolation using CellBender (44) v.2.0 with default settings. In total, 111,806 cells remained.

We performed additional quality control at the individual sample level by calculating the ratio of exonic reads to the total mapped reads using Scrinvex v13 (https://github.com/getzlab/scrinvex). Cells with an exon ratio greater than the 75th percentile + interquartile range are indicative of increased cytoplasmic transcripts and were removed from downstream analyses (6,220 cells). Droplets predicted to contain multiple nuclei as identified by Scrublet (45) were also removed (16,045 cells). Finally, samples were filtered to cells with reads mapped to less than 200 genes (7,864 cells) and cells with more than 5% mitochondrial reads (1,163 cells).

To account for variable complexity between nuclei, counts were normalized to 10,000 unique molecules per nucleus and logarithmized. The gene list was then filtered for highly variable genes (minimum mean 0.0125, maximum mean 3, minimum dispersion 0.5), using a subset of 4,268 genes for cell clustering. Total read count and % mitochondrial reads were regressed out (SCANPY preprocessing *regress_out*) using default parameters, and the data were scaled to a maximum value of 10.

Principal components for the highly variable gene subset was calculated [scanpy.tl.pca(adata, svd_solver='arpack')] and used to correct for batch effects using Harmony (46) (v.0.0.5), considering each sample as a unique batch. Batch-corrected PCs were then used to calculate neighbors [scanpy.pp.neighbors(adata, n_neighbors = 10, n_pcs = 40)] and generate a UMAP [scanpy.tl.umap(adata)]. Cells were clustered using leiden clustering [scanpy.tl.leiden(adata)] at a resolution of 0.4.

### Marker Gene and Cell-Type Identification

To identify marker genes for cell clusters, genes were ranked using a Wilcoxon rank sum test (scanpy *rank_genes_groups*) for each cell-type cluster; and log_2_-fold change (FC), percentage of cells expressing each gene, and area under the receiver operator curve (AUC) scores (SciKit Lean *roc_auc_score*) were calculated for all genes. Genes with an AUC score >0.7 or a log_2_-fold change >0.6 were considered markers of a given cluster.

### Differential Expression Testing

Differentially expressed genes (DEGs) were calculated for each major cell cluster separately using the limma-voom pipeline (Limma v3.48.3). A pseudobulk population was also generated by combining counts across all cell clusters. Counts were first summed per gene within each sample, and low-expressed genes were filtered based on the mean-variance trend (sqrt(residual standard deviation) by average expression) calculated by voom. A linear model was first used to calculate DEGs between sham and band operations within male and female animals separately [model.matrix(∼1 + treatment)]. A second, two-factor model was used to calculate DEGs considering both sex and treatment [model.matrix(∼treatment × sex)]. In all models, a Benjamini–Hochberg false discovery rate (FDR) adjusted *P* value <0.1 was used to identify DEGs. Genes identified as marker genes within other clusters (AUC < 0.7) were removed to exclude potential differential contamination by ambient RNA.

### Compositional Analyses

Cell-type composition was calculated and compared across samples using scCODA (47) (version 0.1.1). scCODA constructs a Markov-Chain Monte Carlo model with Hamiltonian Monte Carlo sampling using cell-type proportions between conditions. We compared cell-type proportions between sham and band operations within female and male animals separately, as well as between female and male banded animals. Differences in cell-type proportions were predicted to be credible via spike-and-slab inclusion probabilities. A degree of composition variability between samples is expected because of differences in nuclei liberation from tissue and successful identification of cells from empty droplets. Cell death and increased fibrosis within the tissue, and low transcriptional complexity, especially within transcript poor cell types, are major drivers of sample composition variability. Our nuclei isolation protocol is designed to mitigate complications with nuclei liberation, whereas the probabilistic cell calling algorithm of CellBender is better able to distinguish cells with lower transcriptional complexity from empty droplets.

### GSEA Pathway Enrichment

Because of the underpowered DEG analysis, we analyzed the gene lists for enriched expression pathways using GSEA preranked (v.6.0.12) (48, 49). Gene lists were ranked according to the *t* statistic previously calculated in the DEG analysis from largest positive value to largest negative value between band and sham animals, considering each cell type and male and female animals separately. Because of conserved gene symbols between the human genome and the feline genome, the ranked gene lists were compared with the human Reactome pathways (v.7.4), comprised of 1,604 gene sets. Any genes that did not have a shared gene symbol with the human genome were removed before analysis. GSEA preranked was run with default parameters (1,000 permutation, weighted scoring, maximum gene set size = 500, minimum gene set size = 15, meandiv normalization mode, and no data set collapse). Reactome pathways and GO Biological Process pathways with a Benjamini–Hochberg FDR adjusted *P* value <0.25 were compared between male and female animals within each cell type.

### Cell-Cell Communication

To investigate communication between cells, CellphoneDB (v.3) (50) was used to detect a significant presence of ligand-receptor pairs between cell types within each condition separately (band and sham operations for both male and female animals). For each analysis, CellphoneDB compares the number of ligand-receptor interacting pairs detected between cell types to the expected number of interactions from randomly permuted cell labels. The analysis was run using normalized count data for all cell types with default parameters (10% threshold for cells expressing ligands and receptors, *P* value = 0.05, 1,000 iterations for generation of null distribution, curated interactions list for human proteins compiled by CellphoneDB from UniProt, Ensembl, PDB, IMEx consortium, and IUPHAR). Interactions within the first two tiers of enrichment were considered significant.

### Statistical Analysis

Data shown are means ± SE or individual data with bar graphs representing means and SE. For echocardiography and compliance, linear mixed-effects models were used to determine and compare predicted mean values at each assessment point (BL and 2 and 4 mo), sex (male, female), and test group (sham, band) differences. In each linear mixed-effects model, time, sex and treatment group were included as fixed effects with interaction terms of time by sex by treatment. For parameters without repeated-measures, two-way analysis of variance (ANOVA) was used to determine significance without interaction term. All analyses were performed using SAS version 9.4 (SAS Institute, Cary, NC). *P* < 0.05 was considered statistically significant.

### Data and Materials Availability

All data, methods, and reagents used during this experiment are available upon request to the corresponding author. Processed single cell data may be downloaded or visualized on the Single Cell Portal of the Broad Institute under study ID: SCP1527. Raw data may be accessed under GEO accession number GSE184328.

## RESULTS

### General Phenotyping

**Figure 2.**
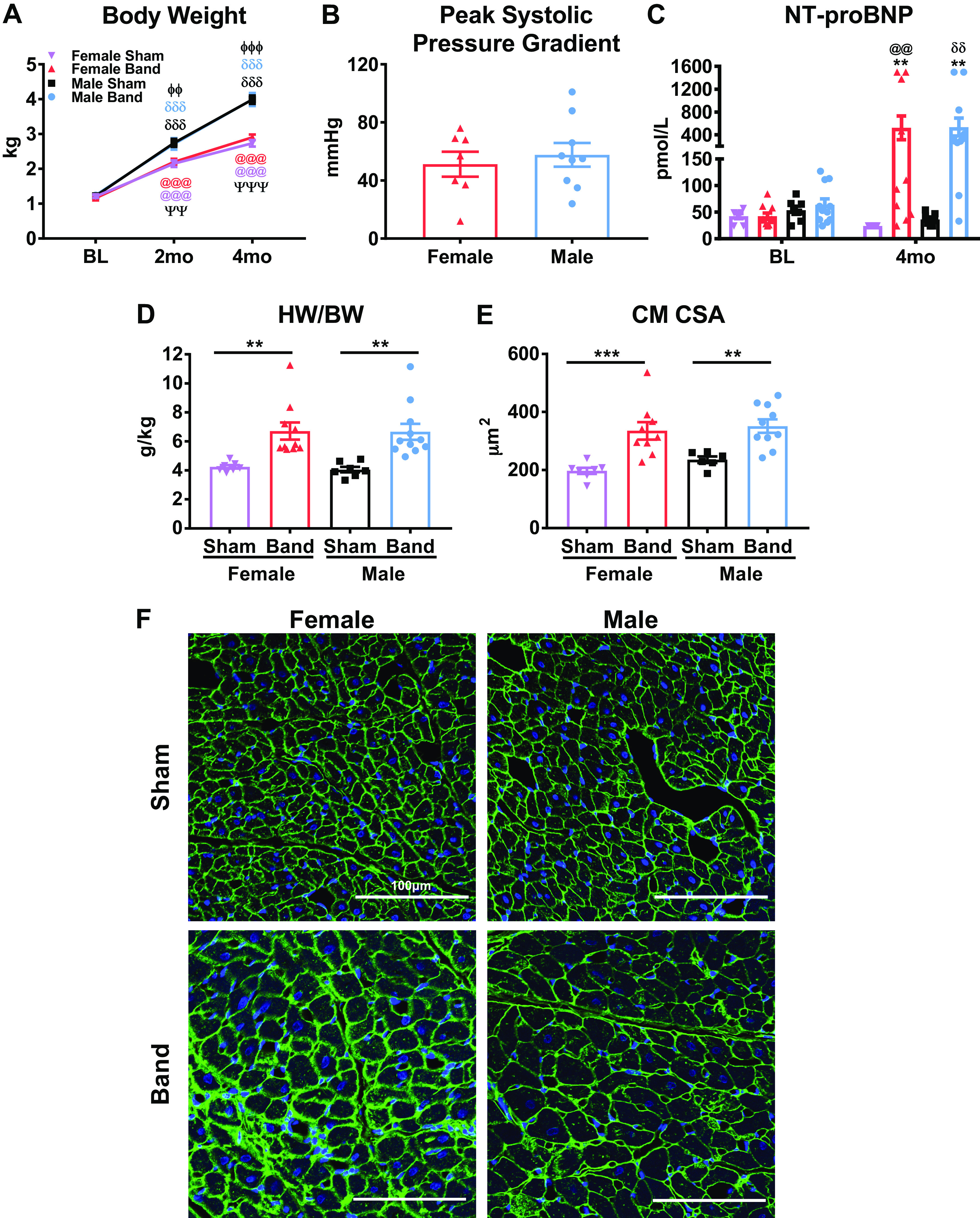
General characteristics. *A*: body weight (BW) was measured at baseline (BL) and 2 and 4 mo postsurgery. *B*: during the terminal study, the peak systolic pressure gradient generated by the aortic band was measuring using fractional flow reserve (FFR). *C*: plasma concentrations of NH_2_-terminal-prohormone B-type natriuretic peptide (NT-proBNP), a heart failure biomarker, were quantified by an independent commercial laboratory. *D* and *E*: cardiac hypertrophy was assessed via heart weight to body weight ratio (HW/BW; *D*) at 4 mo postsurgery, and myocyte hypertrophy was measured histologically using wheat germ agglutinin (WGA) (green, cell membrane) and 4′,6-diamidino-2-phenylindole (DAPI) (blue, nucleus) staining for cardiomyocyte cross-sectional area (CM CSA; *E*). *F*: representative confocal micrographs were taken at ×20 magnification. Scale bar = 100 µm. Data shown are means ± SE (*A*), and individual data with a bar graph representing means and SE (*B–E*). For statistical analysis, significance was determined by using linear mixed-effects models (*A*) and two-way analysis of variance (ANOVA) with interaction (*B–E*). Overall test: *P* = 0.6; *P* < 0.0003 (*C*); *P* < 0.0003 (*D*); and *P* < 0.0002 (*E*). Post hoc test: **P < 0.01, ***P < 0.001, between band vs. sham within the same sex; ΦΦ*P* < 0.01, ΦΦΦ*P* < 0.001 between male band vs. female band; ΨΨ*P* < 0.01, ΨΨΨ*P* < 0.001 between male sham vs. female sham; δδ*P* < 0.01, δδδ*P* < 0.001 between male band or sham vs. BL; @@*P* < 0.01, @@@*P* < 0.001 between female band or sham vs. BL.

There was no significant difference in body weight (BW) between male and female animals (Fig. 2*A*) at the beginning of the study [baseline (BL)]. However, male and female felines grew at different rates and by 2 mo postsurgery, there was a significant BW difference between sexes with females weighing less, independent of band versus sham. By 4 mo postsurgery, this difference in BW between male and females was even more significant, but there was still no difference within each sex between banded and sham animals, documenting that the slow progressive PO did not disrupt normal physiological growth.

There was no significant difference in the peak systolic pressure gradient across the aortic band in female and male animals [female (*n* = 7), 51.29 ± 8.61 mmHg versus male (*n* = 9), 57.78 ± 8.21 mmHg, NS] at 4 mo postsurgery, consistent with comparable PO-induced stress (Fig. 2*B*) in both sexes.

Plasma concentrations of NT-proBNP, a clinically used heart failure biomarker, were measured. At BL, there were no differences between any groups. At 4 mo postbanding, the plasma concentration of NT-proBNP was significantly elevated in both male- and female-banded animals compared with their respective BL levels and to sham animals at 4 mo (Fig. 2*C*). The upper limit of detection for the NT-proBNP test is 1,500 pmol/L, and some animals had concentrations of 1,500 pmol/L and likely exceeded the threshold of the test. Also, there were several banded animals that did not have increased concentrations of NT-proBNP (defined as >100 pmol/L) but still had significant LV hypertrophy.

### Cardiac Hypertrophy

Heart weight-to-body weight ratios (HW/BW) and cardiomyocyte cross-sectional area (CM CSA) were measured as independent indicators of cardiac hypertrophy. Both parameters were significantly increased to similar levels in both sexes of banded animals. There was no significant difference in PO-induced left ventricular hypertrophy (LVH) in male- and female-banded animals (Fig. 2*D*). The HW/BW was increased in both sexes with PO compared with sham animals [female sham (*n* = 7), 4.25 ± 0.12 g/kg vs. female banded (*n* = 10), 6.71 ± 0.59 g/kg, *P* < 0.01; male sham (*n* = 7), 4.06 ± 0.19 g/kg vs. male banded (*n* = 11), 6.66 ± 0.56 g/kg, *P* < 0.01]. CM CSA was significantly increased in male- and female-banded animals, and there were no differences between sexes (Fig. 2*E*). Representative WGA images are shown in Fig. 2*F*. Taken together, these data show that male and female felines develop comparable cardiac hypertrophy when subjected to slow progressive PO.

### Serial Echocardiography

**Figure 3.**
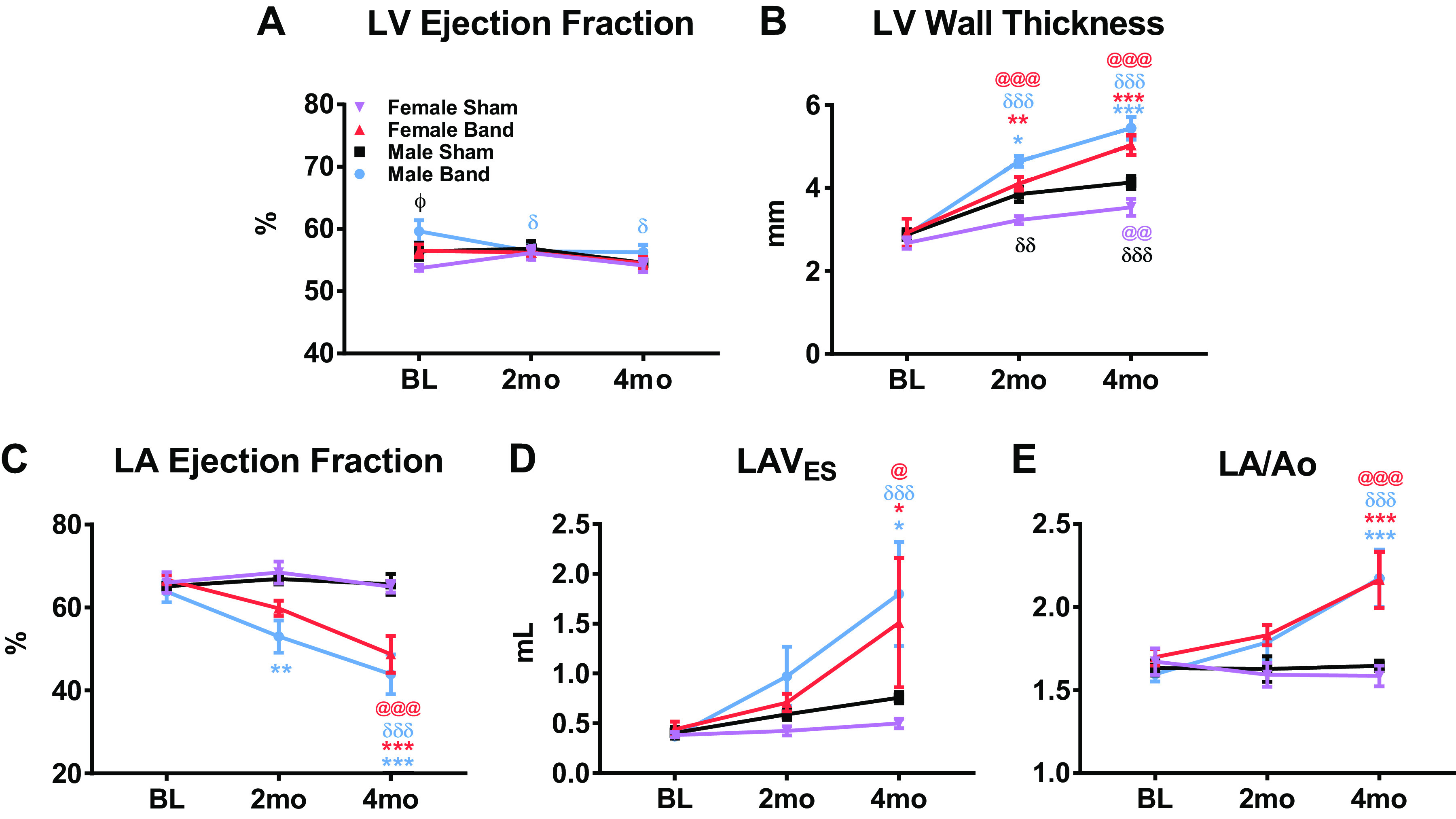
Serial echocardiography. Echocardiography was performed at baseline (BL)1 and 2 and 4 mo postsurgery. *A*: systolic function was assessed via left ventricle (LV) ejection fraction. *B*: LV wall thickness was quantified to examine hypertrophy. *C*: left atrium ejection fraction (LA EF) was measured as a way of measuring LA systolic function. *D* and *E*: two different parameters quantifying LA size were assessed: left atrium end-systolic volume (LAV_ES_; *D*) and ratio of left atrium to aortic root (LA/Ao; *E*). Data shown are means ± SE. Statistical analysis to determine significance was performed using linear mixed-effects models. *P < 0.05, **P < 0.01, ***P < 0.001 between band vs. sham; δ*P* < 0.05, δδ*P* < 0.01, δδδ*P* < 0.001 between male band or sham vs. BL; @*P* < 0.05, @@*P* < 0.01, @@@*P* < 0.001 between female band or sham vs. BL. E and A, peak early and late diastolic transmitral velocities.

Echocardiography (ECHO) was performed at BL and 2 and 4 mo after induction of PO (Fig. 3). LV ejection fraction (EF) was similar across groups at all time points (Fig. 3*A*). However, male-banded animals had a significantly higher LV EF compared with female-banded animals at BL [female banded (*n* = 10), 56.50 ± 1.05% vs. male banded (*n* = 11), 59.64 ± 1.79%, *P* < 0.05]. In addition, male-banded animals had a small but significant decrease in LV EF versus baseline at 2 mo (56.45 ± 1.21 mm, *P* < 0.05) and 4 mo (56.27 m ± 1.24 mm, *P* < 0.05).

Both sexes of banded animals developed increased LV wall thickness at 2 and 4 mo postbanding (Fig. 3*B*), again documenting LV hypertrophy, consistent with the increases in HW/BW and CM CSA. There was no significant difference in LV wall thickness between banded males and females at 4 mo postsurgery [female banded (*n* = 10), 5.03 ± 0.24 mm vs. male banded (*n* = 11), 5.44 ± 0.28 mm, *P* = NS]. Both sexes of sham animals had a significant increase in LV wall thickness at 2 and 4 mo postsurgery, due to the fact that these animals are going through a period of rapid physiological growth.

There was a significant decrease in left atrium (LA) EF from baseline to 4 mo and compared with sham animals in both banded groups [female sham (*n* = 7), 65.00 ± 1.43% vs. female banded (*n* = 10), 48.70 ± 4.44%, *P* < 0.001; male sham (*n* = 7), 65.57 ± 2.40% vs. male banded (*n* = 11), 43.91 ± 4.77%, *P* < 0.001] (Fig. 3*E*). LA EF was not significantly different between sham male and female animals. Female- and male-banded animals both had an increase in both LA end-systolic volume (LAV_ES_) and the ratio of the LA to aortic root dimensions (LA/Ao) (Fig. 3, *D* and *E*), which reached significance within each group at 4 mo postbanding. There was no significant difference in these atrial size and function parameters between banded males and females at 4 mo. These ECHO findings show that both sexes of banded animals develop LV hypertrophy with pathological LA remodeling and LA dysfunction.

### Hemodynamic Measurements

**Figure 4.**
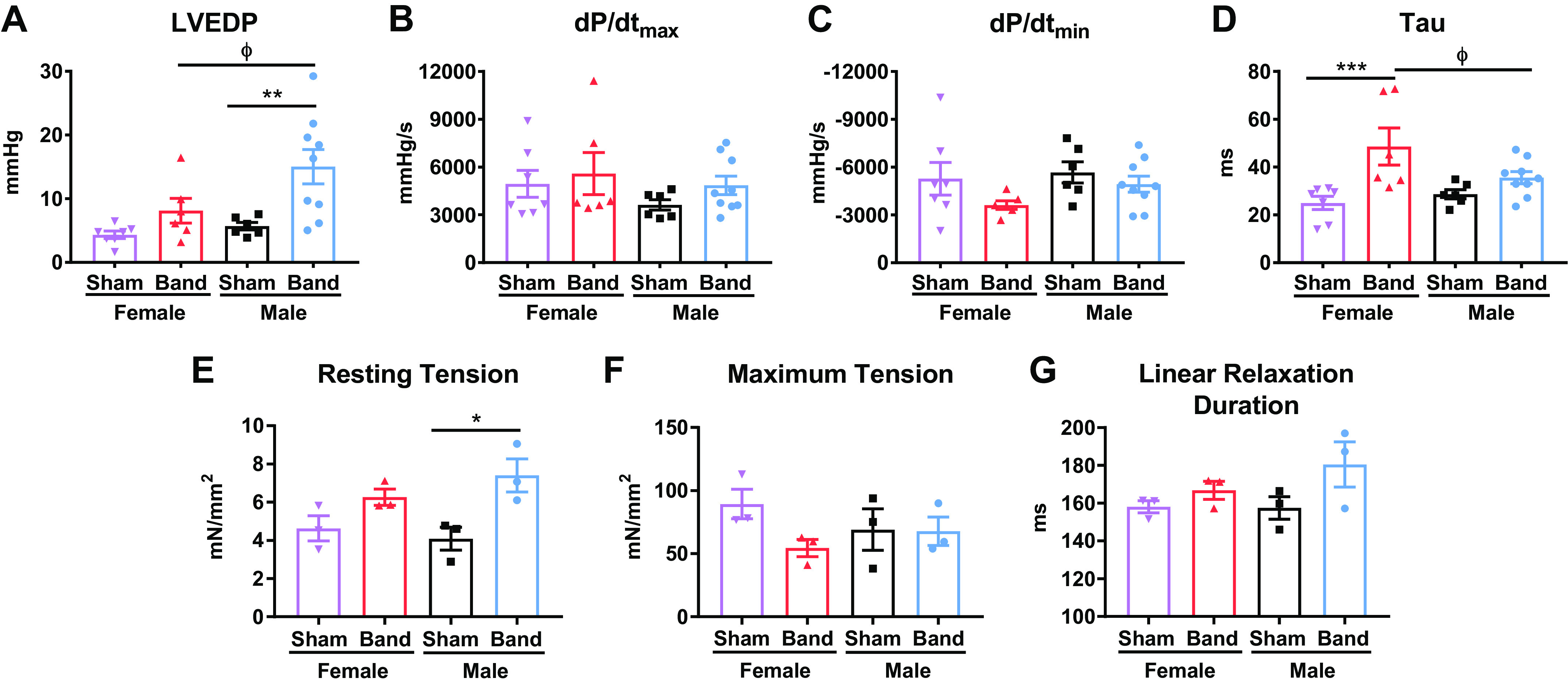
Invasive hemodynamics and sarcomere mechanics. Invasive hemodynamic measurements were measured at 4 mo postbanding. *A–D*: left ventricular end-diastolic pressure (LVEDP; *A*), d*P*/d*t*_max_ (*B*), d*P*/d*t*_min_ (*C*), and τ (*D*) were all assessed. Left ventricle (LV) tissue was collected for sarcomere mechanics studies. *E–G*: myofibril resting tension (*E*), maximum tension (*F*), and linear relaxation duration (*G*) were quantified. *H–J*: correlations between myofibril resting tension and LVEDP (*H*), linear relaxation duration and wall thickness (*I*), and linear relaxation duration and tau (*J*) were evaluated. Data shown are individual data with a bar graph representing means and SE (*A–G*) and fitted linear regression line (*H–J*). Statistical analysis to determine significance was performed using two-way analysis of variance (ANOVA; *A–D*) and Welch’s *t* test (*E–G*) within the same sex. Overall test: *P* = 0.0019 (*A*); *P* = 0.46 (*B*); *P* = 0.25 (*C*); *P* = 0.0032 (*D*). Post hoc test: *P < 0.05, **P < 0.01, ***P < 0.001 between band vs. sham; Φ*P* < 0.05 between male band vs. female band.

Hemodynamic parameters were measured at 4 mo postbanding during terminal studies. Slow progressive PO induced increases in left ventricular end-diastolic pressure (LVEDP) in both sexes. However, the increases in LVEDP in female-banded versus sham animals were not significantly different [female sham (*n* = 7), 4.33 ± 0.57 mmHg vs. female banded (*n* = 6), 8.11 ± 1.91 mmHg, *P* = 0.2065]. The increases in LVEDP in male-banded versus sham animals [male sham (*n* = 6), 5.68 ± 0.60 mmHg vs. male banded (*n* = 9), 15.05 ± 2.70 mmHg, *P* < 0.01] (Fig. 4*A*) were greater than in females and were significantly changed. LVEDP was significantly higher in male-banded versus female-banded animals (*P* < 0.05). There were no significant differences in d*P*/d*t*_max_ or d*P*/d*t*_min_ between any groups (Fig. 4, *B* and *C*).

The LV time constant of isovolumic relaxation (τ) was significantly prolonged in female-banded versus female sham animals [female sham (*n* = 7), 24.98 ± 2.74 vs. female banded (*n* = 6), 48.54 ± 7.73, *P* < 0.001] and female-banded versus male-banded animals (*P* < 0.05) (Fig. 4*D*). There was a trend toward prolongation of τ in male-banded versus male sham animals, but the changes did not reach statistical significance. These results show that PO disrupts the diastolic phenotype of both females and males.

### Sarcomere Mechanics

Myofibrils were isolated from the LV, and their mechanical parameters were measured to examine sex-specific differences in response to banding at the level of the sarcomere. Resting tension was significantly higher in male-banded versus male sham animals. Similar trends were observed in females but were not significantly different in female-banded versus female sham animals [male sham (*n* = 3), 4.09 ± 0.60 mN/mm^2^ vs. male banded (*n* = 3), 7.41 ± 0.87 mN/mm^2^; *P* < 0. 05] (Fig. 4*E*). Maximum isometric tension at maximal calcium (pCa 4.5) was not different in left ventricular myofibrils isolated from male- or female-banded or sham felines (Fig. 4*F*). Myofibrils from both male- and female-banded animals had prolonged linear relaxation compared with sham animals, but these changes were not significant (Fig. 4*G*). Additional myofibril activation and relaxation kinetics are included in Supplemental Fig. S1 (all Supplemental material is available at https://doi.org/10.6084/m9.figshare.c.6163935.v1). These results show that PO caused the development of pathological phenotypes in both females and males, but some changes were less robust in female animals.

### Pulmonary Assessment

**Figure 5.**
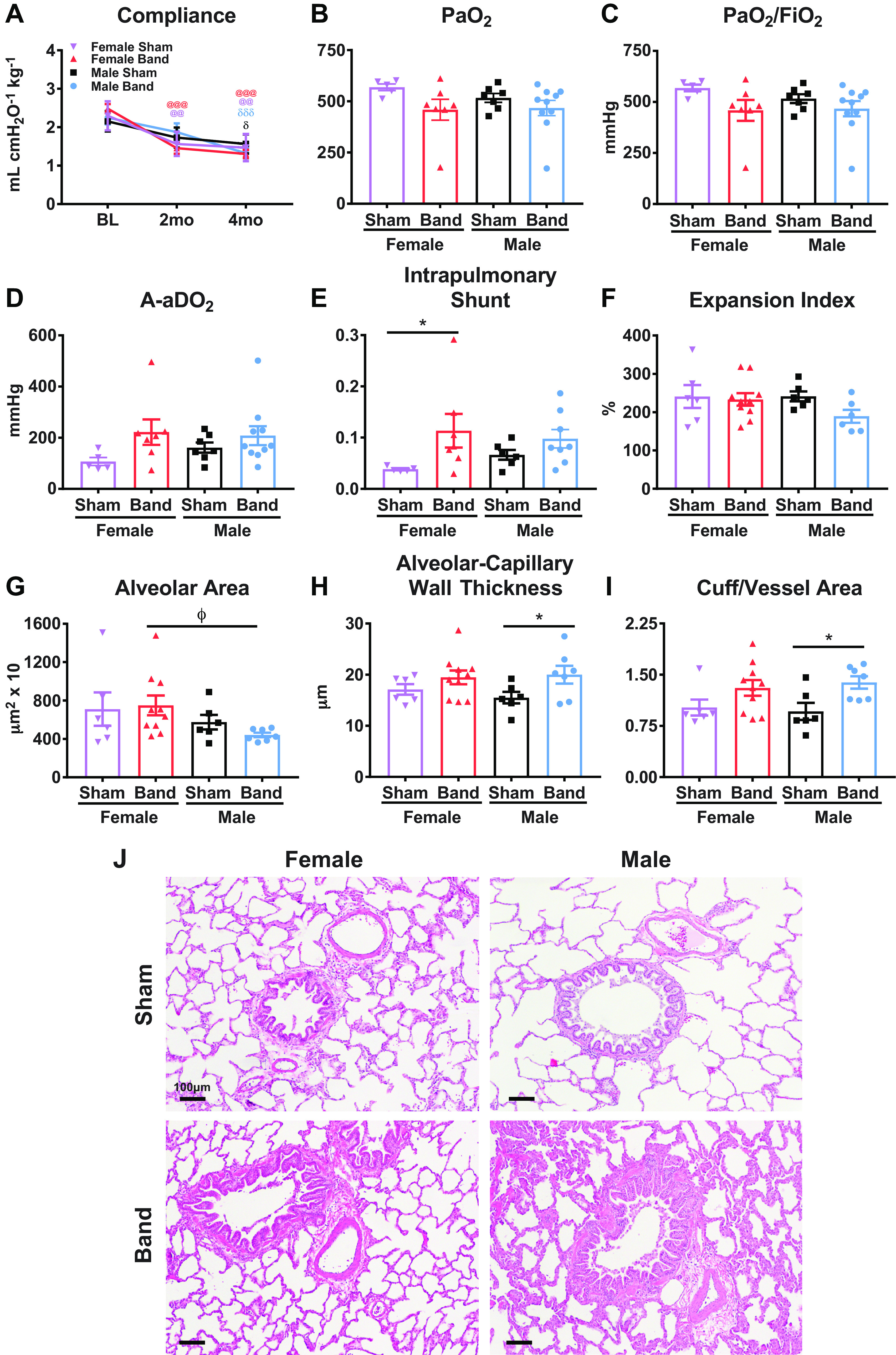
Pulmonary function and histology. *A*: pulmonary function testing was performed at baseline (BL) and 2 and 4 mo postsurgery to assess lung compliance. *B–E*: during terminal studies, partial pressure of oxygen in arterial blood ($Pa_{O_{2}}$; *B*), partial pressure of oxygen in arterial blood to fraction of inspired oxygen ($Pa_{O_{2}}$/${\text{FI}}_{{\text{O}}_{2}}$; *C*), alveolar-arterial oxygen difference (A-aDO_2_; *D*), and pulmonary shunt (*E*) were evaluated. *F–I*: histological analysis of expansion index (*F*), alveolar area (*G*), alveolar-capillary wall thickness (*H*), and ratio of cuff area to alveolar area (*I*) was performed to assess morphological changes. *J*: representative bright-field images of hematoxylin & eosin stain (H&E) stained lung sections. Scale bar = 100 µm. Data shown are means ± SE (*A*) and individual data with a bar graph representing in means and SE (*B–I*). Statistical analysis to determine significance was performed using linear mixed-effects models (*A*) and two-way analysis of variance (ANOVA; *B–I*). Overall test: *P* = 0.24 (*B*); *P* = 0.24 (*C*); *P* = 0.21 (*D*); *P* = 0.12 (*E*); *P* = 0.27 (*F*); *P* = 0.17 (*G*); *P* = 0.13 (*H*); *P* = 0.047 (*I*). Post hoc test: *P < 0.05 between band vs. sham; Φ*P* < 0.05 between male band vs. female band. δ*P* < 0.05, δδδ*P* < 0.001 between male band or sham vs. BL; @@*P* < 0.01, @@@*P* < 0.001 between female band or sham vs. BL.

Pulmonary function testing was performed at BL and 2 and 4 mo postsurgery for serial assessment of lung compliance (Fig. 5*A*). There were no significant differences between groups at BL. Both sexes of banded animals had decreased compliance at 4 mo postbanding (Fig. 5*A*), and there was no significant difference between groups.

Blood gas analyses (BGA) and lung compliance were measured to assess changes in oxygenation and pulmonary function after banding. There were trends toward decreased oxygenation reflected by decreased $Pa_{O_{2}}$ (Fig. 5*B*) and $Pa_{O_{2}}$-to-${\text{FI}}_{{\text{O}}_{2}}$ ratio (Fig. 5*C*) that did not reach statistical significance. Alveolar-arterial oxygen difference (A-aDO_2_) (Fig. 5*D*) was significantly increased in banded animals of both sexes, and intrapulmonary shunting was increased in banded sexes of banded animals compared with their respective shams but only reached significance in females (Fig. 5*E*).

Histological analysis showed that there were no significant changes in expansion index (Fig. 5*F*) in banded males and females but alveolar area (Fig. 5*G*) was significantly reduced in male versus female-banded animals [female band (*n* = 10): 749.90 ± 103.07 vs. male band (*n* = 7): 441.86 ± 23.52, *P* < 0.05] (Fig. 5*G*). Alveolar area was not reduced in banded versus sham females. Male-banded animals had significant increases in alveolar-capillary wall thickness (Fig. 5*H*) and cuff/vessel area (Fig. 5*I*) versus male shams. Female-banded animals showed similar trends in these parameters relative to female shams, but these changes failed to reach significance. Representative images are in shown in Fig. 5*J*. These findings show that PO causes changes in lung structure and function in both females and males but in some instances these changes are less robust in females.

### Extracellular Matrix Remodeling

**Figure 6.**
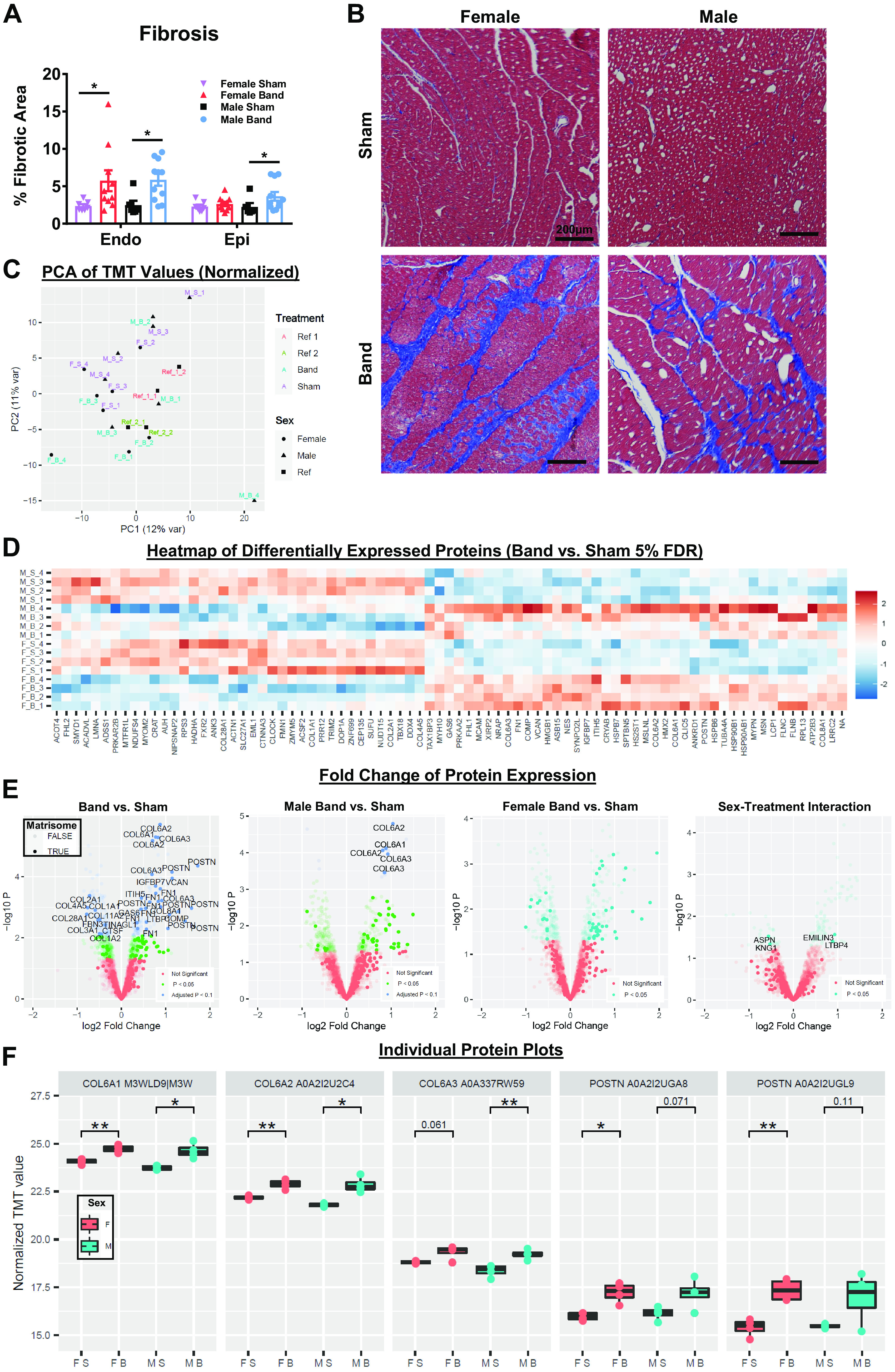
Histological analysis of fibrosis and quantification of extracellular matrix composition by mass spectrometry. Cardiac remodeling and fibrosis was assessed using Masson’s trichrome stained tissue sections. Quantitative mass spectrometry of decellularized left ventricular tissue collected at 4 mo postsurgery was also performed. *A*: percentage of fibrotic area relative to total heart area. *B*: representative brightfield images taken at ×10 magnification of cardiac cross sections to allow for visualization of the endocardium and epicardium; scale bar = 200 µm. *C*: principal component analysis (PCA) reveals the variance in protein distribution. *D*: heat map of differentially expressed proteins. Each row represents data from a single left ventricle (LV), and each column represents an individual protein. Color scale bar represents relative expression of log-transformed normalized expression, with upregulated proteins shown in red and downregulated proteins in blue. *E*: volcano plots show magnitude and significance of proteins altered in all banded cats vs. sham cats, banded male vs. sham male cats, banded female vs. sham female cats, and banded male vs. banded female cats. Proteins identified as belonging to the Naga Matrisome database are highlighted, with cellular components grayed out. Of note, multiple data points are shown for several proteins because of the current redundancy and poor annotation of the feline proteome; therefore, many peptides are mapped to multiple entries. *F*: tandem mass tag (TMT) intensity of type VI collagen and periostin protein expression. F_N, sham female; F_B, banded female; M_N, sham male; M_B, banded male. Data shown are means ± SE. Statistical analysis to determine significance was performed using two-way analysis of variance (ANOVA; *A*). Overall test: Endo, *P* = 0.026; Epi, *P* = 0.11. Post hoc test: *P < 0.05 between banded vs. normal within the same sex. **P < 0.01. FDR, false discovery rate.

Histological analysis of Masson’s trichrome-stained heart tissue was performed to evaluate fibrotic remodeling. Both male- and female-banded animals exhibited significantly greater endocardial fibrosis compared with sham animals [female sham (*n* = 7), 2.38 ± 0.24% vs. female banded (*n* = 10), 5.75 ± 1.40%, *P* < 0.05; male sham (*n* = 6), 2.48 ± 0.60% vs. male banded (*n* = 11), 5.88 ± 0.81%, *P* < 0.05] (Fig. 6*A*). Epicardial fibrosis was also increased in both sexes of banded animals, but these increases were only significant in banded males versus sham animals [male sham (*n* = 6), 2.25 ± 0.50% vs. male banded (*n* = 11), 3.67 ± 0.58%, *P* < 0.05]. Representative bright-field images are in shown in Fig. 6*B*.

Histological approaches for quantifying fibrosis can overlook changes in the composition of the extracellular matrix (ECM) proteins. A more comprehensive evaluation of ECM composition and fibrotic remodeling after aortic banding was performed in decellularized LV tissue using quantitative mass spectrometry (4 banded vs. 4 sham for both sexes). Principal component analysis indicated clear segregations in the constellations of proteins expressed in LV tissues from banded compared with sham animals in both males and females (Fig. 6*C*). Consistent with the cardiac remodeling observed by Masson’s trichrome staining, as depicted in the heat map, there was a substantial increase in the abundance of numerous ECM proteins in LVs of banded male and female felines compared with their respective sham controls; several proteins were also downregulated in banded male and female felines (Fig. 6*D*). The volcano plots in Fig. 6*E* demonstrate the significant modifications in ECM protein expression in LVs of banded male and female felines, with several important ECM components highlighted including type VI collagen and periostin. Many of the alterations in the collective volcano plot appear to be accounted for by banded males, but significant differences are also observed in banded versus sham female animals. Testing for sex-treatment interactions revealed a group of nominally significant proteins that did not pass the multiple testing correction threshold. The effect of aortic banding on fibrotic proteins observed in both females and males is exemplified by quantification of the fibroblast activation markers periostin and type VI collagen (Fig. 6*F*). The mass spectrometry data confirm that ECM protein expression is significantly altered in both male and female felines after banding. A subgroup analysis showed a greater number of differentially expressed proteins in male hearts; however, we did not observe evidence of a sex-biased response.

### Single-Nucleus RNA Sequencing

**Figure 7.**
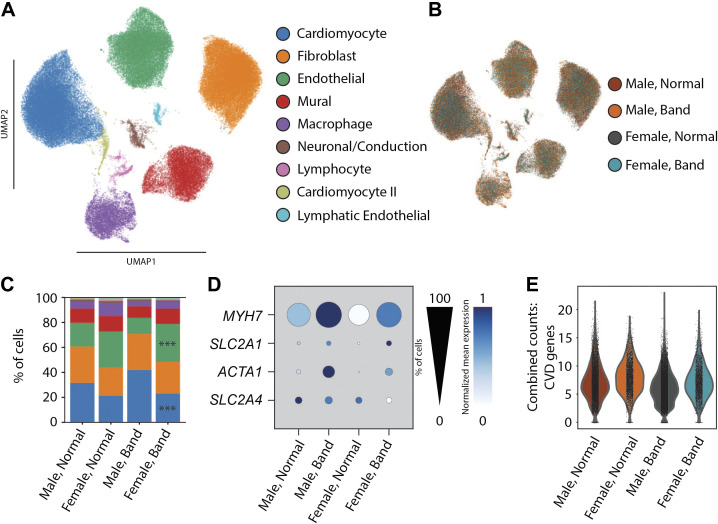
Single-nucleus RNA sequencing. *A*: UMAP projection of 119,670 left ventricular nuclei from four sham male, four banded male, four sham female, and three banded female animals. Clusters are colored by cell type. Colors for each cell type are used throughout. *B*: UMAP colored by sample class displaying representation of each class in each cell type. *C*: bar plot displaying the relative proportion of each cell type in each sample class. ***Significant difference comparing band and sham in that sex. *D*: dot plot displaying the expression of hypertrophic markers in the cardiomyocyte cell class. Size and shade of the dot represent the percentage of cells with nonzero counts and the mean expression, respectively. *E*: violin plot displaying aggregated counts of cardiovascular disease genes (CVD) in cardiomyocytes of each condition.

PO stress could induce cell type-specific transcriptional remodeling in the heart. Bulk RNA sequencing has previously been performed on HFpEF cardiac tissue from both humans (51, 52) and animal models (53–55). This work has laid the fundamental groundwork for defining transcriptomic changes in the disease state, but the results lack specificity in terms of which cell type is undergoing these changes. To determine cell-specific differences in gene expression between sexes and after banding, we performed single-nucleus RNA sequencing in a small group of LV lateral wall samples from sham and banded animals of both sexes (4 vs. 4 for male, 4 vs. 3 for female, respectively). Individual nuclei (119,670) were sequenced with a median of 1,643 unique transcripts per nucleus (Supplemental Table S1). After graph-based clustering, there were nine distinct cell populations (Fig. 7*A*), in line with those observed in larger surveys of humans and model organisms. Sensitive and specific markers for each population are shown in Supplemental Table S2. Although each cell type was observed in hearts from each experimental class (Fig. 7*B*), a proportional expansion of endothelial cells and reduction in cardiomyocytes were uniquely observed in banded females. The trends were preserved in males, but they failed to reach credible thresholds using a Bayesian approach (Fig. 7*C*).

**Figure 8.**
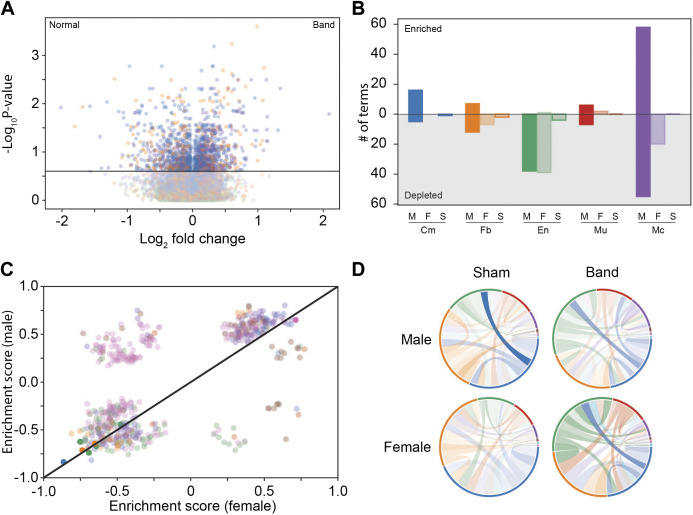
Differential expression analyses of snRNAcsequencing. *A*: volcano plot displaying differentially expressed genes after banding in a combined sex analysis. Colors for this and other panels correspond to those associated with a given cell type in Fig. 7. Horizontal line corresponds to false discovery rate (FDR)-adjusted *P* value threshold of 0.1. *B*: gene set enrichment analysis detailing the number of significant terms by cell type and condition when comparing sham vs. banded animals. M, male-specific terms; F, female-specific terms; S, shared terms; Cm, cardiomyocytes; Fb, fibroblasts; En, endothelial cells; Mu, mural cells; Mc, macrophages. *C*: correlation analysis of enrichment scores for terms that were significant in at least one comparison. Dots with high opacity represent those which were significant in both males and females. *D*: cell-cell communication analysis as performed by CellPhoneDB. Size of colored bands on the outer ring represent the relative proportion of that cell type in that sample class. Saturation of each interconnecting band represents the number of ligand-receptor pairs, which were in the top two tiers of enrichment. Each band is colored by the cell type expressing the relevant ligand.

We next sought to determine differential gene expression within cell types while acknowledging limitations conferred by the relatively small sample size. As pathological hypertrophy is a hallmark of pressure overload, we examined canonical gene markers of hypertrophic programs in cardiomyocytes: *MYH7*, *SLC2A1*, *ACTA1*, and *SLC2A4*. *MYH7*, *SLC2A1*, and *ACTA1* were all increased with PO hypertrophy in both sexes (Fig. 7*D*). As in a recent report of pressure-induced hypertrophy in humans (56), we assessed expression of a “cardiomyocyte disease” metagene comprised of combined counts for *NPPA, NPPB, MYH7, MYH7B, XIRP2, CMYA5, ANKRD1, TNNI3, ACTA1*, and *PFKP*. Similarly to the report in humans, this metagene was upregulated after banding in both males and females (Fig. 7*E*). We also assessed cardiomyocyte expression of Eph receptor tyrosine kinases. Members of the EPHA and EPHB families were reported to have decreased gene expression in human cardiac hypertrophy (56). Interestingly, in the present study, only EPHA7 was highly expressed in all groups (Supplemental Fig. S2). There was very low expression of all other EPHA and EPHB subclasses, irrespective of phenotype. We performed global differential expression analysis with contrasts fit for banding versus sham irrespective of sex (Fig. 8*A*), and banding versus sham in each sex. Full differential expression results can be found in Supplemental Table S3.

To define the cell-specific transcriptional programs that are altered by pressure overload, we next performed ranked gene-set enrichment analysis using the ReactomeDB and GO Biological Process-curated gene sets in the eight most numerous cell types (Fig. 8*B*, Supplemental Tables S4 and S5). In all but endothelial cells, males exhibited markedly more altered gene sets than females. Strikingly, there were relatively few gene sets that were significantly altered in both sexes. We examined whether this phenomenon may be due to a thresholding effect, whereby directionality is preserved with variance in enrichment estimates around the selected FDR cutoff. In more numerous cell types (cardiomyocytes, fibroblasts, endothelial cells, mural cells, and macrophages), this appears to be the case for the majority of terms under a 0.25 FDR, where 91.4% (244/267 terms) share directionality between the sexes. Notably, all altered gene sets in fibroblasts (*n* = 27) shared directionality across the sexes, speaking to a common regulatory response of fibroblasts in response to PO. However, gene sets were regulated in the inverse direction across sexes, speaking to distinct, sex-specific transcriptional programs (Fig. 8*C*) in response to pressure overload. These were particularly notable in macrophage populations, where “heart growth,” “tissue regeneration,” and “leukocyte differentiation” terms were enriched in males but depleted in females.

To estimate changes in cellular communication in response to pressure overload stress, we calculated scores representing the enriched expression of annotated receptor-ligand pairs across all cell types and conditions using CellPhoneDB (Supplemental Table S6). When subsetting for the pairs in the top two ranks of the output, signaling from endothelial cells increased markedly after banding of females (increasing from 14 to 21 pairs), with a lesser number of cell-cell communication pairs in males (from 9 to 17). These included signaling from endothelial PECAM1 to fibroblast CD38, an observation that has not been described in HFpEF. Collectively, these results begin to unravel signaling similarities and differences in males and females in individual cardiac cell types.

## DISCUSSION

Females have an increased prevalence of HFpEF (14). The current study explored the hypothesis that male and female felines respond in a sex-specific fashion when subjected to slow progressive PO. The experiments were performed in a large animal model of slow progressive pressure overload that have been shown to induce key features of HFpEF in male animals (2, 15). The experiments performed compared the cardiopulmonary response to PO at the functional and morphological level of the whole heart and then used state-of-the-art analyses of myofibrils, extracellular matrix, and single-nucleus RNA sequencing to explore the underlying bases for the disease phenotypes in males and females.

There is a clear gap in our knowledge in how both sexes respond to disease stress that leads to HFpEF phenotypes. Although there have been reports describing sex-based differences in rodent models (57–65), studies with larger animals with physiological properties more similar to humans are rare but could define critical sex-specific targets for the treatment of human disease. Our results show that males and females have similar pathological phenotypes when exposed to slow progressive PO. There were some sex-specific differences, but there were more similarities than differences in phenotypic features. In general, females had a similar or less robust response to PO, suggesting PO stress, by itself, does not drive a difference in the HFpEF phenotype for females.

### Cardiac Pathophysiology Caused by PO in Females and Males

The aortic banding procedure generated the same degree of PO stress in males and females. LV EF was preserved (≥ 50%) throughout the entire study in both males and females, and there was no differences in LV diastolic dimensions (no ventricular dilatation). PO induced similar amounts of cardiac hypertrophy in male- and female-banded animals at 4 mo postbanding. LV wall thickness, HW/BW, and CM/CSA were all significantly increased in both sexes after banding. These three independent parameters document equivalent amount of LV and LV myocyte hypertrophy in both sexes.

Left atrial enlargement with reduced atrial EF are signature cardiac changes in HFpEF (66). In the present study, both sexes developed significant atrial remodeling, reflective of elevated LV filling pressures. Both female- and male-banded animals had decreased LA EF at 4 mo postbanding, documenting LA dysfunction. Two independent parameters of LA size, LAV_ES_ and LA/Ao, were both significantly increased in banded animals of both sexes. These data show that LA enlargement and dysfunction are induced to similar extents by PO in both males and females.

Most animal studies that have examined sex-based differences in HFpEF have been performed in rodent TAC models (59–65). The rodent TAC model eventually induces ventricular dilation with reduced ejection fraction rather than a HFpEF phenotype observed in humans (67). The feline model used in this study induces concentric LVH without LV dilation (68–70), as is common in many patients with HFpEF (1). A recent study by Schiattarella et al. (71) reported that male mice fed a high-fat diet and *N*^G^-nitro-l-arginine methyl ester (l-NAME) developed a HFpEF phenotype and found that the spliced X-box binding protein 1 (Xbp1s) signaling pathway was suppressed, which could be a potential mechanism for the development of HFpEF. However, female mice fed the same high-fat diet and l-NAME did not develop a HFpEF phenotype (57), suggesting that perhaps female sex is protective in this model. A study in male and female obese Zucker diabetic fatty/spontaneously hypertensive heart failure F1 hybrid (ZSF1) rats has compared HFpEF phenotypes (72) and found that both sexes of rats developed a comparable cardiac phenotype with diastolic dysfunction, fibrosis, and hypertrophy. The only difference reported (72) was the lack of hyperglycemia in females. The animals used in our study did not have metabolic disturbances, and our results show that in the absence of these disturbances, PO causes a similar pathological cardiac phenotype in male and female animals when assessed using ECHO and other morphological-derived assessments.

### Hemodynamic Evaluation of Cardiac Function following PO

Invasive hemodynamics were used to assess systolic and diastolic function, which is one of the gold standard methods for diagnosing HFpEF (73). Systolic LV function was not different in males and female animals after PO. LV EF was preserved in all animals and d*P*/d*t*_max_, another parameter of systolic function, was not different between groups.

Diastolic filling of the ventricle can be disrupted by alterations of passive stiffness, such as fibrosis, and or by alterations of active relaxation, such as slowing of Ca^2+^ reuptake and slowing of Ca^2+^ unbinding from thin filaments (74, 75). We have previously shown that Ca^2+^ transients are prolonged and SR Ca^2+^ reuptake is slowed in feline myocytes with hypertrophy from PO (68). In the present study, diastolic LV function was assessed using a variety of approaches.

We directly measured LVEDP (Fig. 4*A*). Our results showed that LVEDP was elevated in all banded animals but was not significantly increased in females. This was the most significant hemodynamic difference between males and females observed after PO. Both male- and female-banded animals had significant LV fibrosis with PO, with the magnitude of fibrosis a bit less in females (Fig. 6*A*), which may be responsible for the smaller increase in LVEDP that was observed. Human studies have found no difference in LVEDP between male and female patients with HFpEF (76) and another reported no differences between male and female patients NT-proBNP levels or LV EF measured via ECHO (76).

Tau and d*P*/d*t*_min_ were measured as indices of active relaxation. Tau was prolonged in both males and females but was only significantly prolonged in females. This result suggests that females may have more profound abnormalities in those processes that determine active relaxation that were discussed earlier. Collectively, our data show that PO induces disturbances in diastolic properties of both male and female hearts and there are some minor phenotypic differences between the sexes.

### Myofibril Mechanics Are Impaired with PO

Changes in myofibrillar properties will alter active contraction and relaxation in disease (77). Myofibril mechanical parameters were measured in male and female LV tissues to determine if sex-specific differences were developed in response to PO. Similar to what was observed in the in vivo assessments of active relaxation, myofibril parameters of relaxation were prolonged in both male- and female-banded animals. Likely because of a small sample size, neither group reached statistical significance. Collectively, these results suggest that PO induces similar changes in myofibril function in males and females. Further studies are needed to dissect the contribution of specific mechanisms to the changes in active relaxation that were found in the present studies.

### PO Initiates Abnormal Pulmonary Function

Male-banded animals develop pulmonary hypertension at 4 mo postbanding (2). In the present study, critical features of pulmonary structure and function were compared in male and female animals after PO. Pulmonary compliance was decreased at 4 mo in both sexes, and histological analyses suggest that PO has a greater impact on the male lung expansion pattern (alveolar area) and extravascular fluid accumulation (perivascular cuffs, alveolar-capillary wall thickness) than in females. Interestingly, this structural phenotype is also associated with fluid flux which can present clinically with edema and bronchitis-like symptoms, similar to the clinical presentation of female patients with HFpEF (78, 79). Although both sexes develop a pulmonary phenotype in response to PO, the smaller morphological changes in banded females is likely to be secondary to the smaller increase in LVEDP observed in females. These results show that PO causes changes in lung structure and function in both males and females.

### ECM Remodeling is Similar in Females and Males

Histological analysis of cardiac fibrosis using Masson’s trichrome showed significant endocardial fibrosis in both male and female animals, and there were no differences between the sexes. Females developed a similar fibrotic gradient that we reported in males (2), with a higher percentage of fibrosis in the endocardium that becomes more diffuse and decreases moving toward the epicardium. We previously (2) speculated that this transmural fibrotic gradient develops as a result of PO-induced latent myocardial ischemia and decreases from the endocardium out to the epicardium just like the presentation of the “wavefront phenomenon” that occurs during myocardial infarction with acute coronary occlusion (80). Previous studies have reported a relationship between the content of fibrillar collagen with increased myocardial stiffness (81). Histology gives a general assessment of collagen deposition, but does not provide insight into the composition of the ECM. Travers and coworkers (82) recently performed an in-depth analysis of the ECM using MS, and described the presence of hidden cardiac fibrosis. We used the same ECM MS platform in this study and found an increased abundance of several key proteins in hearts with pressure overload. These changes in ECM protein expression were not different in male and female animals with PO. However, fewer significant proteins were found in females, which is suggestive of a less robust response.

Few studies have addressed sex-based differences in ECM composition in disease. One study reported that females with HFpEF had lower plasma levels of matrix metalloproteinase-3, which is involved in breaking down ECM (83). The importance of these changes are unclear. Collectively, the present results show that extracellular matrix remodeling after induction of PO is similar in males and females.

### Sex-Based Differences in Transcriptional Profile with PO

snRNAseq is a powerful tool for determining changes in gene regulation at the cell-type level, enabling observations in cells that may be masked or missed with bulk RNA sequencing. Recent studies used this platform to determine differences in nuclear gene expression in different cells in the human heart (40, 84). We used this technology to begin a study of transcriptional changes in all cardiac cell types in male and female animals subjected to PO. To the best of our knowledge, this is the first application of snRNAseq on samples from an animal model of HFpEF. Our studies showed that classical “fetal” genes that are known to be altered in pathological hypertrophy induced by PO were changed in cardiac myocyte nuclei from both males and females, but the magnitude of these changes was smaller in females (Fig. 7*D*).

A common finding in all cell types was that a greater number of gene sets with altered expression were found in male animals compared with females (Fig. 7*E*). These results suggest a coordinated transcriptional program that is either specific to, or more prominent in, males than females. The greater magnitude of the effects in males may underlie some of the minor sex-specific phenotypic features differences we observed. In addition, for a minority of terms the directionality of gene regulation is inverse between the sexes. The significance of these finding will need to be explored in future investigations of sex-specific differences linked to pathological remodeling after PO, as well as to other known HFpEF-inducing comorbidities such as metabolic syndrome (71).

Important limitations for this analysis include *1*) the small sample size that limits the statistical power to define additional cell, sex, and PO-specific transcriptional changes and *2*) the lower quality gene annotations for the feline genome when compared with common model organisms and humans, resulting in reduced read mapping metrics and difficulty in extrapolating gene level findings across species lines.

### Spectrum of HFpEF Observed in the Feline Model

Based on the current guideline-based definition of HFpEF, both sexes of animals meet the criteria necessary for a HFpEF diagnosis; preserved LV EF, elevated levels of natriuretic peptides, LV hypertrophy, LA enlargement, and diastolic dysfunction (10, 11). However, not every animal with PO-induced LVH had elevated levels of NT-proBNP, similar to the finding that some patients with HFpEF have normal levels of NT-proBNP (1, 85, 86). A recent study by Verbrugge et al. (87) reported that patients with HFpEF and normal NT-proBNP had increased mortality and heart failure readmissions compared with patients without HF. Shah et al. (88) performed complex unbiased clustering analysis of clinical data (phenomapping) to define different HFpEF phenotypes, and defined three groups of patients: “*1*) younger patients with moderate diastolic dysfunction who have relatively normal BNP; *2*) patients who are obese and diabetic with a high prevalence of obstructive sleep apnea who have the worst LV relaxation; and *3*) older patients with significant chronic kidney disease, electrical and structural myocardial remodeling, pulmonary hypertension, and RV dysfunction.” Our data suggest that the feline model aligns most closely with the first group of patients categorized by Shah et al. (88) in both males and females.

### Limitations

All HFpEF animal models have limitations that require interpretations to be conservative. The limitations of the feline PO model is that animals are young (2 mo of age at the beginning of the study) and have no comorbidities. The young age is essential to the model since growth is the process that leads to slowly evolving aortic constriction (with a fixed band size) and the resulting slow progressive pressure overload that causes LVH without LV dilation. The smaller size of the females versus the males made it technically more challenging to induce the same PO in males and females and to instrument animals for assessing hemodynamic parameters. Future studies in other HFpEF models where comorbidities can be combined should begin to unravel the independent roles of HFpEF comorbidities.

### Conclusions

Slow-progressive PO in this feline model induced similar key features of human HFpEF in both male and female animals. This animal model has its own set of unique limitations but its strengths include the comparable cardiac physiological properties to humans (12, 13). These features could make the model useful for translational research involving HFpEF with PO. Both sexes developed a robust cardiac phenotype, though females had smaller increases in LV filling pressures and lung remodeling than males. snRNAseq studies showed large differences in the amplitude and directionality of cell-specific gene set expression in males and females after PO. These differences may be responsible for the different HFpEF phenotype we observed, which could be tested in mouse models where gene expression can be readily altered in specific cell types (71, 89).

We conclude that PO induces pathological cardiac hypertrophy and fibrosis in males and females, and that females do not have more profound dysfunctional phenotypes. Therefore, PO, by itself is not responsible for differences in the HFpEF phenotype that have been observed in humans (90). We speculate that the unique sex-based differences in cardiac cell gene expression profiles could make females differentially sensitive to specific comorbidities that are linked to human HFpEF.

## SUPPLEMENTAL DATA

10.6084/m9.figshare.c.6163935.v1 Supplemental Figs. S1 and S2 and Tables S1–S6: https://doi.org/10.6084/m9.figshare.c.6163935.v1.

## GRANTS

This study was supported by the National Institutes of Health Grants HL147558 (to S.R.H. and T.A.M.), HL116848, DK119594, HL127240, HL150225 (to T.A.M.), HL140187 (to N.R.T.), K01AG066845 (to K.C.W.), and HL147463 (to J.G.T.); American Heart Association Grant 16SFRN31400013 (to T.A.M.); Ludeman Family Center for Women’s Health Research Pilot Award (University of Colorado Anschutz Medical Campus) (to K.C.W.); CU Consortium for Fibrosis Research and Translation funding (to M.P.Y.L. and E.L.); Bayer Pharma, AG (to M.R.W.); and U.S. Department of Defense-Department of Army, Army Research Laboratory (to M.R.W.).

## DISCLOSURES

T.A.M. is on the scientific advisory boards of Artemes Bio and Eikonizo Therapeutics, received funding from Italfarmaco for an unrelated project, and has a subcontract from Eikonizo Therapeutics related to an SBIR grant from the National Institutes of Health (HL154959). None of the other authors has any conflicts of interest, financial or otherwise, to disclose.

## AUTHOR CONTRIBUTIONS

D.M.E., J.G.T., M.W., E.L., M.P.Y.L., K.C.W., N.R.T., T.A.M., M.R.W., and S.R.H. conceived and designed research; D.M.E., R.M.B., J.E.L., J.G.T., R.D.P., M.L.H., A.R.H.H., G.S., J.P.J., E.L., K.C.W., N.R.T., and M.R.W. performed experiments; D.M.E., J.E.L., J.G.T., R.D.P., M.L.H., H.Z., E.L., K.C.W., N.R.T., and M.R.W. analyzed data; D.M.E., J.G.T., M.L.H., M.W., E.L., M.P.Y.L., K.C.W., N.R.T., M.R.W., and S.R.H. interpreted results of experiments; D.M.E., J.G.T., M.L.H., E.L., M.P.L., K.C.W., and N.R.T. prepared figures; D.M.E., J.G.T., E.L., M.P.L., K.C.W., N.R.T., T.A.M., M.R.W., and S.R.H. drafted manuscript; D.M.E., J.G.T., M.L.H., H.Z., M.W., E.L., M.P.Y.L., K.C.W., N.R.T., T.A.M., M.R.W., and S.R.H. edited and revised manuscript; D.M.E., R.M.B., J.E.L., J.G.T., R.D.P., M.L.H., H.Z., A.R.H.H., G.S., J.P.J., M.W., E.L., M.P.Y,L., K.C.W., N.R.T., T.A.M., M.R.W., and S.R.H. approved final version of manuscript.
